# Reversed-Phase HPLC Characterization and Quantification and Antioxidant Capacity of the Phenolic Acids and Flavonoids Extracted From Eight Varieties of Sorghum Grown in Austria

**DOI:** 10.3389/fpls.2021.769151

**Published:** 2021-11-05

**Authors:** Sofia Speranza, Rebecca Knechtl, Ragnar Witlaczil, Regine Schönlechner

**Affiliations:** Department of Food Science and Technology, Institute of Food Technology, University of Natural Resources and Life Sciences, Vienna, Austria

**Keywords:** sorghum, phenolic acids, flavonoids, RP-HPLC-DAD, Austria, health

## Abstract

Sorghum is raising great interest as a grain for the future, for its agricultural advantages in times of climate change, and for the positive impact of its bioactive compounds on human health. These compounds comprise phenolic acids, in a free, conjugated, and bound form, and flavonoids. The most commonly used extraction methods require high volumes of chemicals and are non-practical when handling many samples at a time. The main aim of this study was to develop a microscale extraction procedure for both phenolic acids and flavonoids to improve yield and diversity, labor time, and chemicals usage. The improved protocols allowed to perform the extraction in 2-ml safe-lock tubes using around 60 times less chemical volume for phenolic acids and 6 times less for flavonoids. In addition, compared to the macroscale method, the microscale approach was effective in extracting a comparable amount of phenolic acids (between 0.99 and 1.57 mg ferulic acid/g) and even a higher quantity of flavonoids (between 1.10 and 2.24 mg ferulic acid/g). With the established methods, phenolic compounds were extracted from eight varieties of sorghum grown in Austria, previously shown to be promising for food processing. In all sorghum varieties, protocatechuic, vanillic, caffeic, syringic, P-coumaric, and ferulic acids were detected in free, conjugated and bound form, with the last being the most abundant. Arsky and Icebergg varieties presented the lowest (922.65 μg/g) and the highest (1,269.28 μg/g) levels of total phenolic acids, respectively, recorded using high-performance liquid chromatography (HPLC). Flavonoids, comprising luteolinidin, apigenidin, naringenin, apigenin, 5-methoxy-luteolinidin (5-MetO-Lut), and 7-methoxy-apigeninidin (7-MetO-Api), were detected in amounts between 27.03 (Kalatur variety) and 87.52 μg/g (Huggo variety). The red varieties, Huggo, Armorik, and Arsky, had the highest antioxidant activity measured as 2,2-Diphenyl-1-picrylhydrazyl (DPPH) [around 5.00 μg Trolox equivalent (TE)/g] and Azino-bis(3-ehtylbenzthiazoline-6-sulfonic acid) (ABTS) (around 3.00 μg TE/g) scavenging capacity for both phenolic acids and flavonoids. Ferric reducing antioxidant power (FRAP) was the highest for the phenolic acids extracted from a white Ggolden variety.

## Introduction

Sorghum is the fifth most cultivated cereal grain in the world and originated from Africa. It is now cultivated all over the world, mostly for animal feed and for ethanol production (Hadebe et al., 2017; Taylor, 2019). Austria accounts only for 2.2% of the European sorghum production (FAOSTAT, 2021), but the production substantially increased over the course of the previous 4 years, from 16,620 tons in 2016 to 29,740 tons in 2018 (FAOSTAT, 2021). In Austria, this cereal grain is still only used for animal nutrition and ethanol production. However, its potential for human consumption and food processing is now being investigated (Rumler et al., 2021). Sorghum is one of the cereal crops that are most resistant to drought as it uses the C4 carbon fixation pathway, an advantageous and efficient photosynthetic process. Consequently, in times of climate change, sorghum is raising great interest as a grain for the future. In addition to the agricultural advantages, sorghum has been shown to positively impact human health, in relation to the high amounts of phytochemicals mainly present in the outer layers of the kernel (Awika and Rooney, 2004). The main compounds of interest are phenolic acids and flavonoids, which are responsible for the characteristic red or yellow color of the grains (Dykes and Rooney, 2006). Phenolic acids in sorghum are wide ranging, with ferulic acid being the most abundant, followed by gallic, O-coumaric, P-coumaric, protocatechuic acids, and others to a lesser extent (Dykes and Rooney, 2006). The majority of these compounds are found in bound and conjugated forms and are linked to the cell wall material. As a result, conventional extraction procedures utilizing only aqueous organic solvents are not enough to release all the phenolics from the complex matrix. Alkali and/or acid extraction steps are needed to break the bonds and liberate the insoluble compounds (Mattila and Kumpulainen, 2002). The better efficacy of one method compared to the other is still controversial as research shows contrasting results in favor for or against both (Verma et al., 2009; Arranz and Calixto, 2010). Still, no standardized and commonly accepted method for the extraction of free and bound phenolic acids adapted for sorghum can be found. In contrast, the literature shows a consensus over the extraction method of flavonoids, using acidified methanol (Awika et al., 2004). However, further optimization of the amount of sample and extraction solvent to be used is still achievable. Flavonoids, comprising mainly anthocyanidins, are also known as 3-deoxyanthocyanins, flavones, and flavanones and are the most interesting class of polyphenols found in sorghum (Dykes and Rooney, 2006). These compounds are of great interest both as food colorants and for their health promoting activity (Girard and Awika, 2018). 3-deoxyanthocyanins, mainly luteolinidin, and apigenidin, are uniquely present in sorghum, conferring to the kernels the characteristic red-yellow color, in the case of pigmented pericarp varieties. The lack of substitution at the C3 position of these anthocyanidins increases their pH and thermal stability. This feature makes these pigments suitable and promising as food colorants (Yang et al., 2014). The color of sorghum extracts is fundamental also in relation to the health properties attributed to this cereal grain as it was shown to positively correlate with their antioxidant capacity (Dia et al., 2016). Among the many therapeutic properties of such extracts, sorghum bioactive compounds were reported to have a significant role in the prevention and treatment of ovarian cancer, and, in particular, naringenin and apigenin extracted from sorghum were shown to synergistically contribute to colon cancer prevention (Yang et al., 2015; Dia et al., 2016).

The aim of this study was to find extraction protocols for phenolic acids and flavonoids adapted for sorghum. The most common methods found in literature were used as a basis for the optimization of phenolic yield and diversity, labor time, and chemicals usage. The objective of this research was also to explore in detail for the first time the polyphenol composition of sorghum varieties grown in Austria. Phenolic acids and flavonoids were characterized using reversed-phase high-performance liquid chromatography with diode array detection (RP-HPLC-DAD), and their antioxidant capacity was investigated by *in vitro* assays.

## Materials and Methods

### Sorghum Varieties and Preparation

Eight tannin-free sorghum varieties, namely Arabesk, Armorik, Arsky, Ggolden, Huggo, Icebergg, Kalatur, and PR88Y92, were grown on an experimental farm in Hörsching (Linz-Land, Austria) and harvested in 2020. The color of the pericarp was white for the varieties Arabesk, Ggolden, Icebergg, Kalatur, and PR88Y92, orange for Arsky, and red for Armorik and Huggo. The kernels were tray-dried after harvest at Saatbau Linz (Leonding, Austria) and further dehusked at Strobl Naturmühle GmbH (Linz-Ebelsberg, Austria). For the analyses, sorghum kernels were freshly ground using the Ultra Centrifugal Mill ZM 200 from Retsch GmbH (Haan, Germany) to pass through a 0.5-mm sieve, and stored refrigerated. Moisture content was determined by using the ICC standard method Nr. 110/1 (ICC, 1976) and ranged between 9.25 and 10.02% (Supplementary Table 1). The results are expressed on a dry matter basis and were carried out three times.

### Chemicals and Standards

Phenolic acids standards comprised trans-ferulic acid, P-coumaric acid, O-coumaric acid, vanillic acid, gallic acid, syringic acid, caffeic acid, and protocatechuic acid. Flavonoid standards included apigenin, apigeninidin chloride, luteolinidin chloride, naringenin, taxifolin, quercetin dihydrate, catechin, and epicatechin. All standards were purchased from Sigma-Aldrich (Steinheim, Germany), Honeywell Fluka^TM^ (Buchs, Switzerland), or Carl Roth GmbH + Co., KG (Karlsruhe, Germany). The Folin–Ciocâlteu reagent was purchased from VWR International (Vienna, Austria), Azino-bis(3-ehtylbenzthiazoline-6-sulfonic acid) (ABTS) from Sigma-Aldrich (Steinheim, Germany), 2,2-Diphenyl-1-picrylhydrazyl (DPPH) and (±)-6-Hydroxy-2,5,7,8-tetra-methylchromane-2-carboxylic acid (Trolox) from Fluka (Buchs, Switzerland), and 2,4,6-Tri(2-pyridyl)-s-triazine (TPTZ) from ACROS Organics (NJ, United States).

### Reflected Light Microscopy of Sorghum Kernels

The microscope Olympus BX51 (Olympus Corporation, Waltham, MA, United States) was used to record the images of the longitudinal section of the kernels. Additional fiber optic light guides were needed as a light source from above. Sorghum kernels from each variety were cut into half with a razor blade and placed on microscope slides. Images were taken at magnification power 4×.

### Phenolic Acids Extraction

**FIGURE 1:**
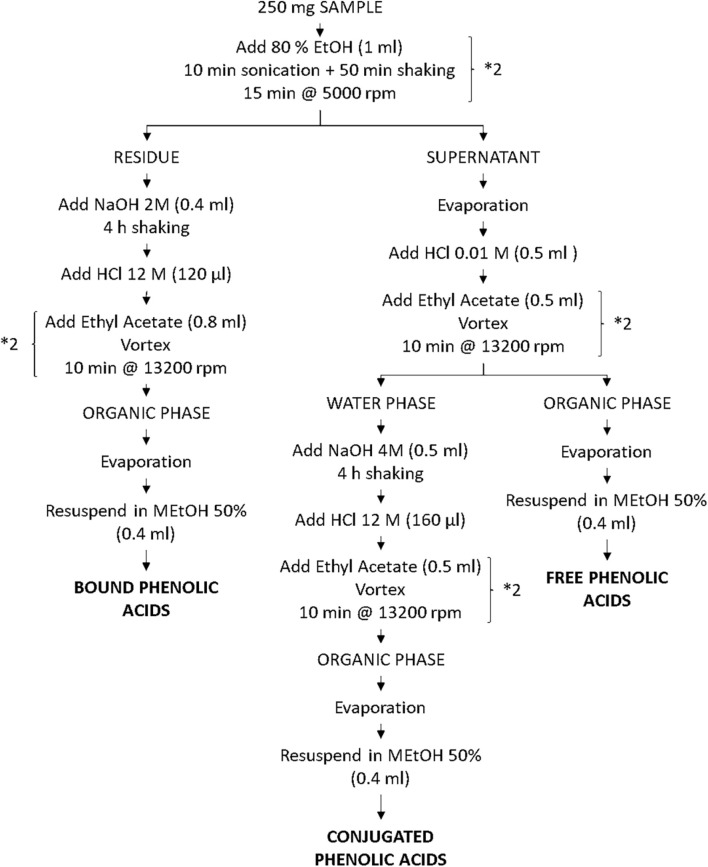
Scheme of the adapted method for the extraction of the free, conjugated, and bound phenolic from sorghum.

For the preliminary trials to adapt the extraction process, a white (Ggolden) and a red (Armorik) sorghum variety were selected. The other six varieties were then extracted with the final adapted extraction method. The extraction procedures for free, conjugated, and bound phenolic acids outlined by Li et al. (2008) and Qiu et al. (2010) were selected as standard protocols and as a basis for method optimization. More details on the trials are mentioned further in section “Results.” The final protocol for the extraction of phenolic acids is shown in Figure 1. To extract free phenolics, 0.25 g of the sample was mixed with 1 ml of 80% ethanol, sonicated for 10 min, and mixed in the dark for 50 min with an orbital shaker. After centrifugation at 2,655 × g for 15 min, the supernatant was collected and the extraction was repeated. The supernatants were combined and dried under vacuum with the Concentrator 5301 from Eppendorf (Hamburg, Germany). The residues were kept for the extraction of bound phenolics. A total of 0.5 ml of 0.01 M HCl was added to the dried supernatant and vortexed. The extraction with 0.5 ml of ethyl acetate was then carried out two times. Samples were centrifuged for 10 min at 18,506 × g, and the organic phases were collected, dried, and resuspended in 0.4 ml of 50% methanol. About 0.4 ml of 4 M NaOH was added to the water phase left from the ethyl acetate extraction. The slurry was mixed and vortexed right after the addition of NaOH to homogenously distribute it. The samples were left shaking for 4 h in the dark. Then, 160 μl of 12 M HCl was added and mixed well. Conjugated phenolic acids were extracted with 500 μl of ethyl acetate two times. Samples were centrifuged for 10 min at 18,506 × g, and the organic phases were collected, dried, and resuspended in 0.4 ml of 50% methanol. Finally, for the extraction of bound phenolic acids, 0.4 ml of 2 M NaOH was added to the residue left from the extraction of the free phenolic. The slurry was mixed and vortexed right after the addition of NaOH. The samples were left shaking for 4 h in the dark. Then, 120 μl of 12 M HCl was added and mixed. Phenolics were extracted with 800 μl of ethyl acetate two times. Samples were centrifuged for 10 min at 18,506 × g, and the organic phases were collected, dried, and resuspended in 0.4 ml of 50% methanol. All samples were filtered through a 0.45-μm polytetrafluoroethylene (PTFE) filter before analyses.

### Flavonoids Extraction

For the preliminary trials to adapt the extraction process, again a white (Ggolden) and a red (Armorik) sorghum variety were selected. The other six varieties were then extracted using the finally adapted extraction method. The extraction procedure detailed by Dykes et al., 2011) with a minor modification was selected as standard protocol and as a basis for method optimization. Briefly, after the first 2 h of extraction and centrifugation, the residue was extracted a second time for 10 min with an additional 10-ml aliquot of HCl/methanol as reported elsewhere (Awika et al., 2004). More details on the trials are mentioned further in section “Results.” The final protocol for the extraction of phenolic acids was as follows: 250 mg of flour was mixed with 1.5 ml of 1 M HCl:methanol (15:85, v:v). The mixture was left shaking for 2 h in the dark and was then centrifuged at 2,655 × g for 15 min. The supernatant was collected separately, and 1.5 ml of 1M HCl:methanol (15:85 v/v) was added to the residue. The extraction was carried out for 10 min followed by centrifugation as mentioned earlier. The supernatants from the two extractions were combined and evaporated. The residue was resuspended in 0.4 ml of 50% methanol. Samples were filtered through a 0.45-μm PTFE filter before analyses.

### Identification and Quantification of Phenolic Compounds With Liquid Chromatography

Filtered samples were analyzed using an HPLC from Shimadzu Corporation (Duisburg, Germany), equipped with a photodiode array detector (Shimadzu SPD-M10A VP). Compounds were separated using a reversed phase C18 Eurospher II column (100 mm × 3 mm i.d., 3 μm) with an integrated precolumn, from KNAUER Wissenschaftliche Geräte GmbH (Berlin, Germany). The flow was set to 0.7 ml/min, and the column temperature was maintained at 50°C. The injection volume for both standards and samples was 10 μl. Around 0.025% trifluoroacetic acid (TFA) in water was used as mobile phase A, and 0.025% TFA in acetonitrile as mobile phase B. Phenolic acids were separated with the following gradient: 0–25 min: 2–15% B; 25.5–30 min: 99% B; and 30.5–36 min: 2% B. Gallic, protocatechuic, vanillic, syringic, and O-coumaric acids were detected at 268 nm, caffeic, P-coumaric, FAs at 320 nm (Supplementary Figure 1). The gradient for the separation of flavonoids was: 0–25 min: 5–25% B; 25–35 min: 25–28% B; 35.5–40 min: 99% B; and 40.5–45 min: 5% B. Catechin, epicatechin, taxifolin, and naringenin were detected at 280 nm, luteolinidin chloride and apigenidin chloride at 480 nm, and apigenin at 340 nm (Supplementary Figure 2). The standards were first solubilized in methanol. Dilutions for the calibration curve were done in mobile phase A.

Compounds in the samples were identified based on the retention time of the standards, and the quantification of each compound was accomplished using calibration curves based on the peak areas of the standards. The identification of 5-methoxy-luteolinidin (5-MetO-Lut) and 7-methoxy-apigeninidin (7-MetO-Api) was based on UV spectra found in the literature (Taleon et al., 2014; Su et al., 2017), and the quantification was determined using the calibration curves from luteolinidin chloride and apigenidin chloride. The resulting concentration was multiplied by the molecular weight correction factor (MWCF) (0.93 for both compounds) (Chandra et al., 2001). For the calculation of the MWCF, the m/z value of 306.7, 290.7, 285, and 269 for luteolinidin chloride, apigenidin chloride, 5-MetO-Lut, and 7-MetO-Api, respectively, were used (Wu and Prior, 2005).

### Total Phenolic Content

For the determination of total phenolic content (TPC), the Folin–Ciocâlteu method was carried out as outlined by Herald et al., 2012). In a 96-well plate, each well was filled with 75 μl of water and 25 μl of either sample or standard, and 25 μl of Folin–Ciocâlteu reagent (diluted 1:1 (v/v) with water). After 6 min in the dark, 100 μl of 75 g/L Na_2_CO_3_ was added to each well. The absorbance at 765 nm was measured with a spectrophotometric microplate reader (set to shake for 60 s before reading) after 90 min of rest in the dark. Trans-ferulic acid dissolved in the sample matrix at a concentration between 250 and 25 mg/L was used as standard. The results were expressed as mg FA/g sample.

### Antioxidant Activity Assays

To assess the antioxidant capacity of phenolic compounds in sorghum, ABTS, DPPH, and ferric reducing antioxidant power (FRAP) assays were carried out on the samples extracted using the adapted method. Trolox was used as standard, and the results were expressed as μmol Trolox equivalent (TE)/g.

Azino-bis(3-ehtylbenzthiazoline-6-sulfonic acid assay was performed as described by Re et al. (1999) and Lee et al. (2016). The ABTS radical cation solution was prepared by mixing ABTS and sodium persulfate with water at the concentrations of 7.46 and 2.44 mM, respectively. The solution was incubated in the dark for 16 h and stored in the freezer until use. For the analysis, the ABTS radical solution was diluted with ethanol until reaching an absorbance of 0.7–0.8 at 734 nm. Then, 190 μl of this solution was mixed with 10 μl of each sample in a 96-well plate. After 1 h, a decrease of absorbance was measured at 734 nm using a microplate reader.

The DPPH assay was adapted from Fukumoto and Mazza (2000) and Herald et al. (2012). DPPH solution (150 μM) was prepared in 80% methanol. Sample and standard solutions (25 μl) were filled in a 96-well plate. Next, 200 μl of the DPPH solution was added, mixed, covered, and allowed to react in the dark for 6 h. The absorbance was read at 517 nm using a microplate reader.

The FRAP assay was carried out by following the procedure of Benzie and Strain (1996). The FRAP reagent was prepared by mixing 25 ml of acetate buffer, pH 3.6, 2.5 ml TPTZ solution in 0.04 M HCl, and 2.5 ml FeCl_3_^∗^6H_2_O solution. About 10 μl of the sample was added to each well, with 30 μl water, followed by 300 μl of the FRAP reagent, previously warmed to 37°C. Samples were incubated for 8 min at 37°C, and the absorbance was measured at 593 nm.

### Statistical Analyses

Statistical data analysis was performed using Statgraphics Centurion 19 (Statpoint Technologies, Inc., Warrenton, VA, United States). One-way ANOVA and Fisher’s least significant tests were carried out with a significance level of α = 0.05. Pearson product moment correlations were also performed between pairs of variables to assess the statistical significance of correlation (*p* < 0.01). The results are expressed as mean ± SD from triplicate measurements. Significant differences between the results are reported using different superscript letters.

## Results

**FIGURE 2:**
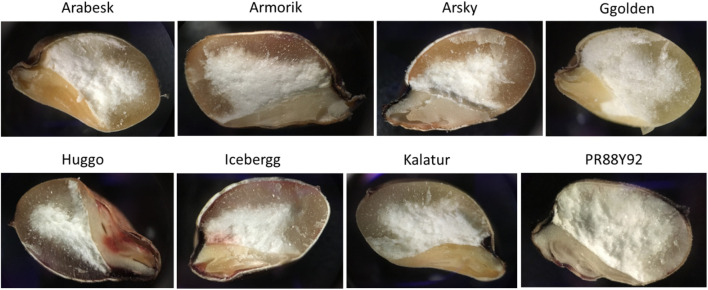
Microscope (Olympus BX51) shows the images of the longitudinal section of the kernels of the eight sorghum varieties used in this study. Magnification power: 4×. The average kernel length was between 3.9 and 4.7 mm (Supplementary Table 1).

Images of the sections of sorghum kernels were taken using the microscope to show the diversity among varieties (Figure 2). Three kernels per variety were analyzed, and only one of the triplicates is shown. Between the replicates of the same variety, a great variation was observed in endosperm and germ color, size, and shape. No significant difference was detected between the eight varieties as a consequence of a great variation within the same variety. The major and only discriminant between them was the red or white pericarp, not appearing in the microscope pictures.

### Phenolic Acids Extraction Optimization

**FIGURE 3:**
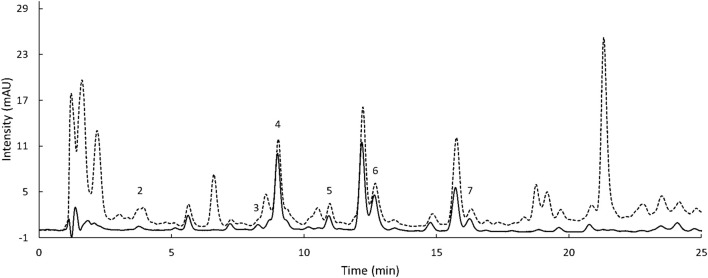
Chromatogram of the free phenolic fraction of Armorik variety, extracted with ethanol 80%, before ethyl acetate extraction (Method 5, dash line) and after (Method 3, solid line), recorded at 268 nm. 2: protocatechuic acid, 3: vanillic acid, 4: caffeic acid, 5: syringic acid, 6: P-coumaric acid, and 7: ferulic acid. The injection volume was 10 μl.

For the extraction of the phenolic acids from grains, various methods found in literature share the main steps and reagents selection. Namely, a first extraction with either aqueous ethanol or methanol is usually followed by alkaline hydrolysis with NaOH. Among the numerous procedures reported, the extraction outlined by Qiu et al. (2010) was selected as standard protocol (Method 1). A summary of the phenolic acids extraction trials is reported in Table 1. This, likewise the majority of the other methods, utilizes unnecessarily high amounts of reagent solutions, specifically, 80 ml of 80% methanol, 40 ml of 4 M NaOH, and 210 ml of ethyl acetate per sample. In contrast, for the screening of phenolic acids in wheat, Li et al. (2008) carried out an unusual microscale procedure (Method 2), reducing the amount of solutions to 3, 0.4, and 1.6 ml, respectively. Such a microscale approach would significantly reduce chemical waste and labor time and would ease the process as already reported by Zavala-López and García-Lara (2017). Therefore, the first extraction trials aimed to compare Methods 1 and 2 (Table 2). As the recovery of free phenolic acids with Method 2 was significantly lower compared to Method 1 (0.20 and 0.11 mg FA/g for Method 2 for Armorik and Ggolden, respectively, vs. 1.65 and 0.91 mg FA/g for Method 1), the extraction time with ethanol 80% was adjusted from 10 min to 1 h (Method 3). This modification increased slightly the free phenolic acids recovery, to 0.23 and 0.14 mg FA/g (Table 2). To further investigate the difference in TPC between the macroscale and microscale approaches, ethanol 80% (Method 3) and methanol 80% (Method 4) were compared for extraction efficiency. The increased yield with methanol was only significantly higher for Armorik (Table 2). Additionally, no difference in phenolic acids diversity between the two solvents was recorded using the HPLC (Supplementary Figure 4). Therefore, since methanol is a toxic substance, ethanol was still the solvent of choice. Another major difference between Methods 1 and 2 was the additional ethyl acetate extraction of the free phenolic acids of Method 2. To evaluate the contribution of this additional step on the recovery, Methods 3 and 4 were carried out omitting the additional ethyl acetate extraction (Methods 5 and 6, respectively). As expected, TPC, after the ethyl acetate extraction was significantly lower as only the phenolic acids soluble in ethyl acetate, were extracted (Table 2). However, the chromatograms from Method 3 showed a better peak separation and baseline noise (Figure 3). Additionally, as both the conjugated and bound fractions were also extracted with ethyl acetate, this step was maintained for the free phenolic acids to obtain three consistent extracts. A further modification to Method 3 involved the extraction of the conjugated phenolic acids. According to Method 3, alkaline hydrolysis was carried out on the free phenolic extract after evaporation. By doing so, the extract contains both free and conjugated phenolic acids (Li et al., 2008). For the adapted method (Figure 1), to release the esterified phenolic acids, alkaline hydrolysis was performed on the water phase left over after the free phenolic extraction with ethyl acetate (Chandrasekara and Shahidi, 2011). This allowed to only extract the conjugated fraction without the free phenolics already liberated in the ethyl acetate phase. This adjustment also allowed to start from the same sample and to obtain three fractions, whereas Method 3 required to start with two different subsamples for the extraction of the same fractions. With regard to the bound phenolic acids extraction, the protocol was kept as it was in the original method as the difference in TPC values was considered acceptable (Table 2).

**TABLE 1:** Summary of extraction trials for free, conjugated, and bound phenolic acids (PA) performed on Armorik and Ggolden varieties.

Method 1	(Qiu et al., 2010)
Method 2	(Li et al., 2008)
Method 3	(Li et al., 2008): 1 h instead of 10-min extraction with ethanol 80%
Method 4	(Li et al., 2008): Method 3 with methanol 80% instead of ethanol 80%
Method 5	Method 3 without ethyl acetate extraction for free phenolic acids
Method 6	Method 4 without ethyl acetate extraction for free phenolic acids

**TABLE 2:** Total phenolic content (TPC) of the free, conjugated, and bound PA from different extraction methods.

		Method 1	Method 2	Method 3	Method 4	Method 5	Method 6
FP	Armorik	1.65 ± 0.03^e^	0.20 ± 0.01^a^	0.23 ± 0.02^a^	0.29 ± 0.04^b^	1.01 ± 0.07^c^	1.29 ± 0.03^d^
	Ggolden	0.91 ± 0.06^d^	0.11 ± 0.00^a^	0.14 ± 0.00^b^	0.14 ± 0.01^b^	0.63 ± 0.02^b^	0.74 ± 0.02^c^
CP	Armorik	0.25 ± 0.03^b^	0.18 ± 0.01^a^	0.20 ± 0.00^a^			
	Ggolden	0.16 ± 0.02^a^	0.15 ± 0.01^a^	0.17 ± 0.00^a^			
BP	Armorik	0.81 ± 0.04^c^	0.66 ± 0.01^a^	0.71 ± 0.02^b^			
	Ggolden	1.17 ± 0.26^b^	0.86 ± 0.04^a^	0.86 ± 0.04^a^			

*The results are expressed as mean value ± SD (n = 3). The superscript indicates that the methods used are significantly different (different letters). All results are expressed on dry matter, in mg FA/g.*

### Flavonoids Extraction Optimization

**FIGURE 4:**
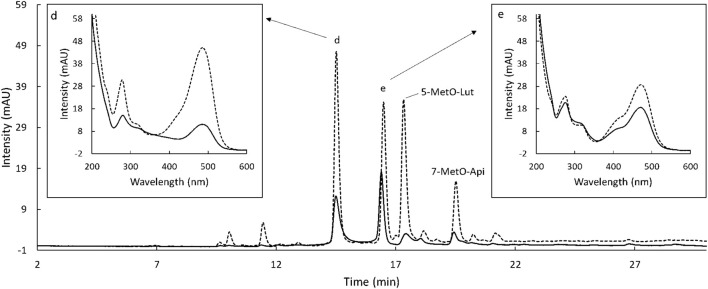
High-performance liquid chromatograph (HPLC) chromatogram of flavonoids extracted from Armorik with acetone 70% (solid line) and with hydrochloric (HCl)/MetOH (dash line) recorded at 480 nm. d: luteolinidin-chloride, e: apigenidin-chloride; f: naringenin, and g: apigenin. In addition, the UV spectra of peak d (left) and e (left), corresponding to the samples extracted with acetone 70% (solid line) and with HCl/MetOH (dash line).

The extraction of flavonoids from sorghum was first carried out by following the procedure outlined in Dykes et al. (2011) (Method A). A summary of the extraction trials is reported in Table 3. To reduce solvent usage and facilitate the procedure allowing more samples to be extracted at the same time, the amount of sample was decreased from 1 g (Method A) to 250 mg and the amount of HCl/methanol from 10 to 1.5 ml (Method B). This modification led to the same yield of extracted flavonoids, even higher for Ggolden (Table 4). To further improve the extraction yield and shorten the extraction time, the effect of a sonication step was assessed. Sonication has been often reported as the preferred method of extraction of bioactive compounds from plant material as it enhances the yield (Muñiz-Márquez et al., 2013; Dzah et al., 2020). Samples were extracted two times with HCl/methanol for 10 min in a sonication bath (Method C) or in an orbital shaker (Method D). No significant differences were obtained by using sonication instead of only shaking (Table 4). Also, increasing the extraction rounds from 2 to 4 and increasing the sonication time to 20 and 30 min did not positively affect the extraction yield (results not shown). In addition, the combination of sonication (15 min) with shaking (45 min) followed by a second extraction (10 min sonication + 10 min shaking) (Method E) compared with 1 h of only shaking followed by a second 20-min extraction (Method F) did not enhance the extraction, nor the compound variety recorded using the HPLC (Supplementary Figure 5). Finally, trials with acetone 70% were also carried out as previously tested by Awika et al. (2004) and Bueno-Herrera and Pérez-Magariño (2020). A 2-h extraction with acetone 70% followed by a second extraction lasting 10 min (Method G) also did not increase the flavonoid content of the extracts (Table 4). Moreover, the structure of the 3-deoxianthocyanidins extracted using this procedure was altered as previously reported (Figure 4; Awika et al., 2004). The UV spectra of luteolinidin and apigenidin after the extraction with acetone presented much lower absorption maxima and at a slightly different wavelength, suggesting changes in the compound structure. Therefore, acetone was not considered a proper solvent for the extraction of flavonoids. After these numerous trials, Method B was considered to be an optimal procedure for the extraction of flavonoids from sorghum.

**TABLE 3:** Summary of extraction trials for flavonoids performed on Armorik and Ggolden varieties.

Method A	(Dykes et al., 2011) with a second 10-min extraction (1 g of the sample in 10 ml)
Method B	Method A with 250 mg of the sample in 1.5 ml
Method C	Two 10-min sonication-aided extractions (250 mg of the sample in 1.5 ml)
Method D	Two 10-min extractions with shaking (250 mg of the sample in 1.5 ml)
Method E	15-min sonication + 45-min shaking extraction followed by 10-min sonication + 10-min shaking extraction (250 mg of the sample in 1.5 ml)
Method F	1-h shaking extraction followed by 20-min shaking extraction (250 mg of the sample in 1.5 ml)
Method G	Method B with acetone 70% instead of HCl/methanol

**TABLE 4:** A comparison between TPC of the flavonoids extracted from Ggolden and Armorik with the different extraction methods.

	Method A	Method B	Method C	Method D	Method G
Armorik	2.32 ± 0.09^c^	2.31 ± 0.04^c^	2.00 ± 0.08^a,b^	1.94 ± 0.02^a^	2.10 ± 0.03^b^
Ggolden	1.31 ± 0.04^c^	1.37 ± 0.03^d^	1.14 ± 0.01^b^	1.13 ± 0.03^b^	1.08 ± 0.01^a^

*The results are expressed as mean value ± SD (n = 3). The superscript indicates, for each sample, that the methods used are significantly different (different letters in the same row). All results are expressed on the dry matter in mg FA/g.*

### High-Performance Liquid Chromatography Identification and Quantification of Phenolic Compounds

**FIGURE 5:**
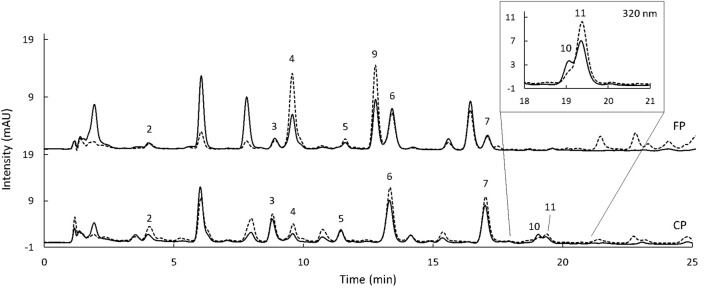
HPLC chromatogram of free (FP, upper chromatogram) and conjugated (CP, lower chromatogram) phenolic acids extracted from Kalatur (solid line) and Huggo (dash line) was recorded at 268 nm. Peaks 10 and 11 (CP) is shown also at 320 nm (from 18 to 21 min). 2: protocatechuic acid, 3: vanillic acid, 4: caffeic acid, 5: syringic acid, 6: P-coumaric acid, 7: ferulic acid, and 9, 10, 11: peaks tentatively identified (see section “Discussion”). Injection volume: 10 μl.

**FIGURE 6:**
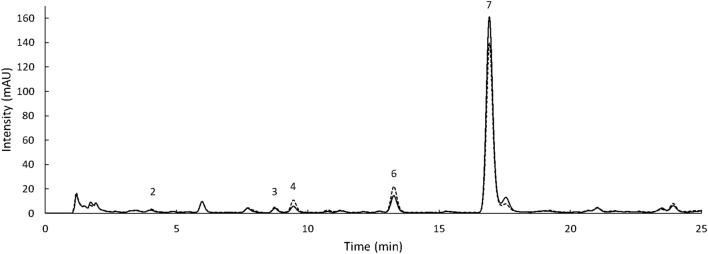
HPLC chromatogram of the bound phenolic acids extracted from Kalatur (solid line) and Huggo (dash line) was recorded at 268 nm. 2: protocatechuic acid, 3: vanillic acid, 4: caffeic acid, 5: syringic acid, 6: P-coumaric acid, and 7: ferulic acid. Injection volume: 10 μl.

**FIGURE 7:**
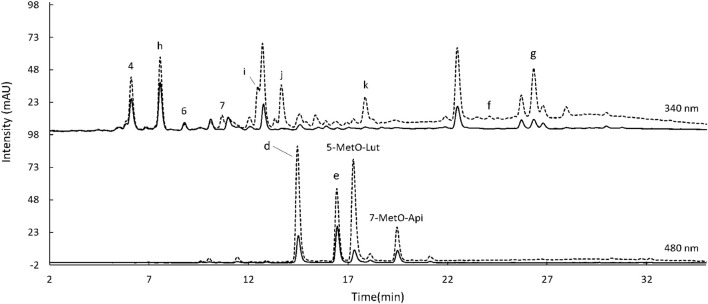
HPLC chromatogram of flavonoids extracted from Kalatur (solid line) and Huggo (dash line) recorded at 340 nm (above) and 480 nm (below). 4: caffeic acid, 6: P-coumaric acid, 7: ferulic acid, d: luteolinidin-chloride, e: apigenidin-chloride; f: naringenin, g: apigenin, and h–k: unknown peaks, tentatively identified (see section “Discussion”). Injection volume: 10 μl.

Separate methods were selected for phenolic acids and flavonoids as otherwise overlapping between the standards occurred. The standards were selected based on the compounds reported in sorghum. Gallic and O-coumaric acids were not detected in any of the phenolic acids fractions (Figures 5, 6). Likewise, catechin, epicatechin, and taxifolin were not present in any of the flavonoid extracts (Figure 7). To show the HPLC chromatograms of the sample extracts, Kalatur and Huggo varieties were chosen as representative for all the eight varieties as these varieties contained approximately the lowest and the highest amounts of phenolic compounds.

Phenolic acids were mainly found in their bound form, with ferulic acid being the most abundant, reaching a concentration of around 1.00 mg/g, approximately eight times higher compared to free and conjugated forms (Table 5). Syringic acid was the only compound not present in bound form. Contrary to all the other phenolic acids, the concentration of caffeic acid was approximately three to four times higher in a free form compared to a conjugated form. Caffeic acid was also the most abundant phenolic acid in the free phenolic acid fraction, whereas ferulic acid was the most abundant in the conjugated and bound fractions. Huggo, Armorik, and Icebergg varieties presented the highest content of free, conjugated, and bound phenolic acids, respectively (31.70, 37.73, and 1,224.16 μg/g). Overall, Icebergg variety presented the highest concentration of total phenolic acids, 1,269.28 μg/g and Arsky the lowest, 922.65 μg/g (Supplementary Table 2).

**TABLE 5:** Content of free, conjugated, and bound PA in the eight varieties of sorghum calculated from the peak areas of the high-performance liquid chromatography (HPLC) chromatograms.

	Protocatechuic acid	Vanillic acid	Caffeic acid	Syringic acid	P-Coumaric acid	Ferulic acid	Total PA
**Free phenolic acids (μ g/g)**							
Arabesk	0.71 ± 0.09^c,d^	1.28 ± 0.04^d^	8.05 ± 0.18^a^	1.34 ± 0.00^b^	6.21 ± 0.12^d^	3.10 ± 0.07^a,b^	20.70 ± 0.39^b^
Armorik	*n*.*d*.	0.66 ± 0.03^a^	16.69 ± 0.03^e^	1.87 ± 0.03^d^	5.08 ± 0.06^b^	3.35 ± 0.05^b,c^	27.65 ± 0.15^d^
Arsky	0.31 ± 0.01^a^	1.54 ± 0.08^e^	11.42 ± 0.38^c^	0.97 ± 0.07^a^	5.13 ± 0.25^b^	8.02 ± 0.32^e^	27.40 ± 1.08^d^
Ggolden	*n*.*d*.	0.93 ± 0.02^b^	9.01 ± 0.28^b^	1.37 ± 0.07^b^	5.03 ± 0.15^b^	3.26 ± 0.07^b,c^	19.60 ± 0.57^b^
Huggo	0.87 ± 0.23^d^	1.29 ± 0.06^d^	17.97 ± 1.04^f^	1.58 ± 0.08^c^	5.65 ± 0.66^c^	3.57 ± 0.46^c^	31.70 ± 1.64^e^
Icebergg	0.64 ± 0.03^b^	1.09 ± 0.14^c^	14.50 ± 0.55^d^	1.51 ± 0.07^c^	4.86 ± 0.29^b^	3.05 ± 0.24^a,b^	25.50 ± 0.89^c^
Kalatur	1.13 ± 0.02^e^	1.68 ± 0.06^f^	9.13 ± 0.44^b^	1.39 ± 0.06^b^	7.23 ± 0.24^e^	4.26 ± 0.13^d^	24.82 ± 0.90^c^
PR88Y92	0.66 ± 0.06^b^	0.75 ± 0.03^a^	9.12 ± 0.31^b^	1.00 ± 0.06^a^	3.76 ± 0.18^a^	2.76 ± 0.12^a^	18.05 ± 0.70^a^
**Conjugated phenolic acids (μ g/g)**							
Arabesk	2.78 ± 0.19^b^	3.49 ± 0.27^b,c^	2.45 ± 0.20^a^	2.03 ± 0.21^d^	8.49 ± 0.75^c,d^	12.30 ± 1.04^a,b^	31.53 ± 2.47^b,c^
Armorik	3.65 ± 0.30^d^	3.58 ± 0.08^b,c,d^	3.00 ± 0.12^a,b^	2.59 ± 0.01^e^	8.12 ± 0.39^b,c,d^	16.79 ± 1.03^d^	37.73 ± 1.20^d^
Arsky	1.52 ± 0.06^a^	3.27 ± 0.16^a,b^	2.64 ± 0.13^a^	1.10 ± 0.05^a^	6.06 ± 0.31^a^	11.31 ± 0.40^a^	25.91 ± 1.07^a^
Ggolden	2.24 ± 0.17^b,c^	4.17 ± 0.18^e^	2.46 ± 0.15^a^	2.21 ± 0.05^d^	7.48 ± 0.17^b,c^	15.33 ± 0.20^c,d^	33.89 ± 0.59^c,d^
Huggo	2.80 ± 0.43^c^	4.04 ± 0.40^d,e^	3.78 ± 1.17^b^	1.68 ± 0.10^b,c^	8.89 ± 0.36^d^	13.88 ± 0.70^b,c^	35.07 ± 2.93^c,d^
Icebergg	1.86 ± 0.47^a,b^	3.22 ± 0.31^a,b^	2.15 ± 0.31^a^	1.54 ± 0.22^b^	7.64 ± 1.31^b,c^	11.71 ± 1.54^a^	28.13 ± 4.09^a,b^
Kalatur	1.97 ± 0.90^a,b^	3.76 ± 0.45^c,d,e^	2.85 ± 1.19^a,b^	1.95 ± 0.32^c,d^	8.06 ± 0.86^b,c,d^	12.66 ± 1.26^a,b^	31.25 ± 4.26^b,c^
PR88Y92	1.62 ± 0.06^a,b^	2.84 ± 0.10^a^	2.03 ± 0.04^a^	1.68 ± 0.04^b,c^	7.04 ± 0.27^a,b^	12.72 ± 0.08^a,b^	27.93 ± 0.18^a,b^
**Bound phenolic acids (μ g/g)**							
Arabesk	6.43 ± 0.34^a,b^	6.43 ± 0.67^a^	39.18 ± 13.47^a,b,c^	n.d.	71.98 ± 5.62^b,c^	821.01 ± 56.54^a^	945.03 ± 73.52^a,b^
Armorik	7.22 ± 0.16^b,c^	7.01 ± 0.18^a,b^	49.39 ± 2.47^c^	n.d.	61.51 ± 2.91^a,b^	952.54 ± 29.89^a,b^	1077.67 ± 34.93^b,c^
Arsky	6.18 ± 0.54^a,b^	8.27 ± 0.69^b,c^	26.46 ± 2.21^a^	n.d.	48.87 ± 6.45^a^	779.56 ± 159.62^a^	869.34 ± 167.05^a^
Ggolden	5.56 ± 1.59^a^	8.48 ± 1.49^b,c^	38.88 ± 7.93^a,b,c^	n.d.	50.42 ± 7.04^a^	955.19 ± 95.81^a,b^	1058.53 ± 100.07^a,b,c^
Huggo	8.16 ± 1.03^c,d^	11.96 ± 1.51^d^	43.06 ± 6.16^b,c^	n.d.	76.11 ± 11.68^c,d^	840.98 ± 155.80^a^	980.28 ± 175.56^a,b^
Icebergg	9.22 ± 0.82^d^	9.02 ± 0.73^c^	43.86 ± 8.09^b,c^	n.d.	72.47 ± 2.38^b,c^	1089.58 ± 73.56^b^	1224.16 ± 72.41^c^
Kalatur	6.04 ± 0.93^a,b^	9.26 ± 1.09^c^	27.98 ± 9.66^a^	n.d.	53.06 ± 13.98^a^	854.19 ± 126.01^a^	950.53 ± 100.62^a,b^
PR88Y92	7.17 ± 0.04^b,c^	9.23 ± 0.35^c^	32.62 ± 1.07^a,b^	n.d.	87.68 ± 4.66^d^	898.88 ± 82.61^a^	1035.58 ± 86.68^a,b,c^

*Total PA was calculated as the sum of the six compounds. The results are expressed as mean value ± SD (n = 3). The superscript indicates, for each compound, that samples are significantly different (different letters, in the same column). All results are expressed on dry matter. n.d.: not detected.*

The compounds mostly present in the flavonoid extracts were the 3-deoxyanthocyanidins luteolinidin and apigenidin (Table 6). The red varieties, Armorik, Arsky, and Huggo, had the highest content of luteolinidin, between 18.69 and 32.69 μg/g, which is the compound responsible for the red-orange color (Kambal and Bate-Smith, 1976). The flavone apigenin and the flavanone naringenin were also detected in all sorghum varieties, but at lower concentrations, between 1.14 and 11.02 μg/g, respectively (Table 6). Generally, the concentration of the integrated flavonoids varied from 27.03 μg/g for Kalatur to 87.52 μg/g for Huggo.

**TABLE 6:** The content of flavonoids in the eight varieties of sorghum was calculated from the peak areas of the HPLC chromatograms.

	Luteolinidin	Apigenidin	Naringenin	Apigenin	5-MetO-Lut	7-MetO-Api	Total flavonoid
Arabesk	15.14 ± 0.58^c^	24.57 ± 1.06^f^	1.94 ± 0.10^b^	3.58 ± 0.12^d^	10.27 ± 0.37^b^	11.65 ± 0.79^d^	57.74 ± 2.45^d^
Armorik	18.69 ± 1.08^d^	13.39 ± 0.72^d^	7.12 ± 0.91^d^	1.70 ± 0.79^a^	13.47 ± 0.41^c^	6.08 ± 0.15^b^	44.47 ± 1.69^c^
Arsky	30.74 ± 1.32^e^	19.74 ± 0.94^e^	11.02 ± 0.63^e^	3.08 ± 0.22^c,d^	22.40 ± 1.35^d^	9.15 ± 0.48^c^	70.30 ± 2.39^e^
Ggolden	11.69 ± 0.26^b^	7.64 ± 0.37^a^	1.14 ± 0.06^a,b^	2.21 ± 0.10^a,b^	9.41 ± 0.18^b^	4.12 ± 0.13^a^	28.04 ± 0.96^a^
Huggo	32.69 ± 0.33^f^	21.17 ± 0.67^e^	5.28 ± 1.03^c^	9.64 ± 0.53^e^	26.69 ± 1.57^e^	9.35 ± 0.16^c^	87.52 ± 1.96^f^
Icebergg	17.28 ± 0.70^d^	9.44 ± 0.52^b,c^	0.84 ± 0.17^a^	2.54 ± 0.09^b,c^	15.10 ± 0.72^c^	5.45 ± 0.31^b^	37.43 ± 1.83^b^
Kalatur	8.76 ± 0.43^a^	10.44 ± 0.16^c^	1.14 ± 0.06^a,b^	2.50 ± 0.17^b,c^	4.92 ± 0.28^a^	4.02 ± 0.19^a^	27.03 ± 1.02^a^
PR88Y92	11.39 ± 2.25^b^	8.84 ± 1.45^a,b^	1.73 ± 0.59^a,b^	2.40 ± 0.25^b^	8.32 ± 2.87^b^	4.39 ± 0.38^a^	29.51 ± 2.02^a^

*Total flavonoid was calculated as the sum of the six compounds. The results are expressed as mean value ± SD (n = 3). The superscript indicates that the samples are significantly different (different letters in the same column). All the results are expressed on the dry matter in μg/g.*

### Antioxidant Activity Assays

Huggo, Armorik, and Arsky varieties presented the highest TPC for free phenolic acids (0.31, 0.21, and 0.25 mg FA/g, respectively) and for conjugated phenolic acids (0.11, 0.09, and 0.09 mg FA/g, respectively) (Table 7). For the bound phenolic acids fraction, Icebergg variety had the highest TPC, 0.96 mg FA/g. ABTS and DPPH assay results correlated positively (*r* > 0.60, *p* < 0.01) with TPC values. FRAP had a statistically significant correlation only for free phenolic acids. Overall, the sum of the TPC of free, conjugated, and bound phenolic acids was the highest in Huggo, Arsky, and Armorik, with 1.57, 1.36, and 1.19 mg FA/g, respectively. Also, for the total sum of phenolic acids, the TPC correlated positively with ABTS and DPPH antioxidant capacity (*r* > 0.82, *p* < 0.01). Ggolden variety presented in total the highest value for FRAP, 4.36 μmol TE/g. Armorik, Arksi, and Huggo varieties presented also the highest TPC for the flavonoid extracts, 2.24, 2.02, and 1.96 mg FA/g, respectively (Table 8). Also, the DPPH, ABTS, and FRAP values for these three varieties were the highest among the eight varieties.

**TABLE 7:** TPC and antioxidant activity of the free, conjugated and bound PA extracted from the eight varieties of sorghum and of the sum of the three fractions.

	TPC (mg FA/g)	DPPH (μ mol TE/g)	ABTS (μ mol TE/g)	FRAP (μ mol TE/g)
**Free phenolic acids**				
Arabesk	0.12 ± 0.03^b^	0.84 ± 0.07^c^	0.60 ± 0.05^b^	0.50 ± 0.03^a,b^
Armorik	0.23 ± 0.02^c^	1.14 ± 0.03^d^	0.81 ± 0.04^c^	0.76 ± 0.03^c^
Arsky	0.25 ± 0.01^c^	1.20 ± 0.01^d^	0.92 ± 0.06^d^	0.73 ± 0.02^c^
Ggolden	0.11 ± 0.01^b^	0.72 ± 0.04^a,b^	0.42 ± 0.01^a,b^	0.54 ± 0.03^b^
Huggo	0.31 ± 0.02^d^	1.36 ± 0.01^e^	1.21 ± 0.10^e^	0.80 ± 0.07^c^
Icebergg	0.11 ± 0.01^a,b^	0.74 ± 0.06^b^	0.61 ± 0.24^a,b^	0.48 ± 0.07^a,b^
Kalatur	0.11 ± 0.01^a,b^	0.67 ± 0.01^a^	0.41 ± 0.04^a^	0.45 ± 0.01^a^
PR88Y92	0.09 ± 0.01^a^	0.70 ± 0.04^a,b^	0.32 ± 0.03^a^	0.46 ± 0.02^a^
**Conjugated phenolic acids**				
Arabesk	0.07 ± 0.01^b^	0.42 ± 0.05^a^	0.56 ± 0.06^a^	0.35 ± 0.03^b,c^
Armorik	0.09 ± 0.01^c^	0.68 ± 0.01^c^	0.69 ± 0.03^c^	0.39 ± 0.02^c^
Arsky	0.09 ± 0.01^d^	0.52 ± 0.05^b^	0.67 ± 0.07^b^	0.34 ± 0.02^a,b,c^
Ggolden	0.06 ± 0.00^a,b^	0.52 ± 0.07^b^	0.60 ± 0.01^b^	0.37 ± 0.01^b,c^
Huggo	0.11 ± 0.00^e^	0.68 ± 0.03^c^	0.72 ± 0.00^c^	0.36 ± 0.05^b,c^
Icebergg	0.05 ± 0.01^a,b^	0.45 ± 0.05^a,b^	0.58 ± 0.03^a,b^	0.31 ± 0.02^a,b^
Kalatur	0.06 ± 0.01^a,b^	0.42 ± 0.04^a^	0.50 ± 0.02^a^	0.28 ± 0.07^a^
PR88Y92	0.05 ± 0.00^a^	0.43 ± 0.03^a^	0.55 ± 0.03^a^	0.33 ± 0.03^a,b,c^
**Bound phenolic acids**				
Arabesk	0.75 ± 0.03^a^	2.92 ± 0.03^a^	1.61 ± 0.06^a,b^	2.77 ± 0.19^a^
Armorik	0.77 ± 0.04^a,b^	3.16 ± 0.12^b^	1.95 ± 0.03^a^	2.84 ± 0.06^a,b^
Arsky	0.85 ± 0.04^a,b,c^	3.22 ± 0.09^b,c^	1.86 ± 0.03^a,b^	2.90 ± 0.19^a,b^
Ggolden	0.87 ± 0.07^b,c,d^	3.39 ± 0.11^c^	2.02 ± 0.02^b^	3.45 ± 0.19^c^
Huggo	0.94 ± 0.10^c,d^	3.31 ± 0.14^b,c^	2.03 ± 0.10^b^	3.17 ± 0.29^b,c^
Icebergg	0.96 ± 0.04^d^	3.32 ± 0.11^b,c^	2.09 ± 0.02^b^	3.39 ± 0.16^c^
Kalatur	0.87 ± 0.02^b,c,d^	3.32 ± 0.14^b,c^	1.83 ± 0.05^b^	2.97 ± 0.26^a,b^
PR88Y92	0.87 ± 0.03^b,c,d^	3.23 ± 0.01^b,c^	1.90 ± 0.01^a,b^	3.35 ± 0.10^c^
**Free + conjugated + bound phenolic acids**				
Arabesk	0.99 ± 0.10^a^	4.19 ± 0.10^a^	2.78 ± 0.13^a^	3.62 ± 0.23^a^
Armorik	1.22 ± 0.01^b^	4.98 ± 0.11^d^	3.46 ± 0.02^c^	3.99 ± 0.08^b,c^
Arsky	1.36 ± 0.07^c^	4.94 ± 0.13^d^	3.46 ± 0.10^c^	3.97 ± 0.19^b,c^
Ggolden	1.12 ± 0.09^a,b^	4.63 ± 0.06^c^	3.05 ± 0.03^b^	4.36 ± 0.22^d^
Huggo	1.57 ± 0.14^d^	5.36 ± 0.17^e^	3.96 ± 0.21^d^	4.33 ± 0.22^d^
Icebergg	1.19 ± 0.04^b^	4.51 ± 0.11^b,c^	3.27 ± 0.27^b,c^	4.18 ± 0.19^a^
Kalatur	1.09 ± 0.02^a,b^	4.41 ± 0.17^b^	2.74 ± 0.04^a^	3.69 ± 0.23^a,b^
PR88Y92	1.04 ± 0.04^a^	4.35 ± 0.01^a,b^	2.77 ± 0.01^a^	4.14 ± 0.11^c,d^

*The results are expressed as mean value ± SD (n = 3). The superscript indicates that the samples are significantly different (different letters). All the results are expressed on dry matter.*

**TABLE 8:** TPC and antioxidant activity of the flavonoids extracted from the eight varieties of sorghum.

	TPC (mg FA/g)	DPPH (μ mol TE/g)	ABTS (μ mol TE/g)	FRAP (μ mol TE/g)
Arabesk	1.16 ± 0.02^a,b^	5.14 ± 0.01^a^	1.82 ± 0.04^c^	2.87 ± 0.04^b^
Armorik	2.24 ± 0.02^d^	5.84 ± 0.05^d^	2.52 ± 0.04^d^	4.91 ± 0.10^f^
Arsky	2.02 ± 0.06^c^	5.63 ± 0.03^c^	2.84 ± 0.06^e^	4.39 ± 0.27^e^
Ggolden	1.16 ± 0.01^a,b^	5.29 ± 0.05^b^	1.55 ± 0.05^a,b^	3.05 ± 0.13^b,c^
Huggo	1.96 ± 0.03^c^	5.68 ± 0.07^c^	2.78 ± 0.09^e^	4.33 ± 0.08^e^
Icebergg	1.16 ± 0.03^a,b^	5.33 ± 0.07^b^	1.52 ± 0.05^a^	3.12 ± 0.10^c,d^
Kalatur	1.10 ± 0.05^a^	5.15 ± 0.03^a^	1.71 ± 0.26^b,c^	2.59 ± 0.07^a^
PR88Y92	1.20 ± 0.04^b^	5.32 ± 0.04^b^	1.59 ± 0.02^a,b^	3.27 ± 0.07^d^

*The results are expressed as mean value ± SD (n = 3). The superscript indicates that the samples are significantly different (different letters). All the results are expressed on dry matter.*

## Discussion

In this study, free, conjugated, and bound phenolic acids and flavonoids were extracted using a microscale adapted method from Austrian grown sorghum. Compared to the macroscale method, the microscale approach was effective in extracting a comparable amount of phenolic acids and even a higher quantity of flavonoids. The free phenolic acid yield obtained with the adapted method was still much lower than the macroscale alternative (around 80 and 70% lower for Armorik and Ggolden, respectively) due to the additional ethyl acetate extraction step present in the adapted procedure. Despite achieving a lower yield, this step was important for improving the HPLC chromatographic resolution and for consistency with the other two fractions. The microscale approach was not only effective but also efficient in terms of the amount of chemicals used, labor time, and convenience. Overall, the improved extraction protocols allowed to use around 60 times less chemical volume for the extraction of phenolic acids and 6 times less for flavonoids. In addition, the procedure could be carried out in 2-ml safe-lock tubes. In this way, more samples could be analyzed simultaneously, and the handling and storing were of greater ease. These microscale methods were, therefore, suitable for the screening of phenolic compounds in sets of multiple samples for analytical purposes as not much sample is required for further analyses, such as HPLC and antioxidant assays.

With the established methods, the eight sorghum varieties grown in Austria were analyzed for their phenolic acid and flavonoid profile and content. For the optimization of the HPLC methods for phenolic acids and flavonoids, several columns (differing in length, pore size, and stationary phase), gradients, and temperatures were assessed. The most important factors to achieve the best chromatographic resolution were using a 100 mm C18 column and selecting 50°C as temperature (instead of a longer column, 150 or 250 mm, and 35°C, the most commonly used parameters found in the literature). In addition, the methods were made as short as possible without compromising the resolution. The best results were obtained with runs of 35 and 45 min for phenolic acids and flavonoids, respectively, whereas methods found in literature applied usually at least 1 h (Li et al., 2008; Bueno-Herrera and Pérez-Magariño, 2020). From the HPLC results, the Icebergg variety had the greatest amount of phenolic acids as a sum of the free, conjugated, and bound, amounting to 1,269.28, μg/g, followed by Arabesk and Armorik. The concentration of phenolic acids found in the eight varieties of Austrian grown sorghum was in the same range as previously reported data for sorghum (Hahn et al., 1983; Girard and Awika, 2018). The lowest amount, 922.65 μg/g, was recorded for Arsky variety. The TPC range matched with the values calculated from the chromatographic areas as the TPC of the sum of the three phenolic acids fractions varied between 0.99 (Arabesk variety) and 1.57 mg FA/g (Huggo variety). However, in contrast to the HPLC results, the Huggo and Arsky varieties presented the highest scores with 1.57 and 1.36 mg FA/g. This difference could be due to the fact that with the HPLC only a selection of peaks, for which a standard was available, in the case of this study only of six, was considered for the calculations. Meanwhile, TPC considers all compounds interacting with the Folin–Ciocâlteu reagent, comprising both phenolic and non-phenolic compounds and other reducing substances. Thus, it is less specific and selective than the HPLC. For the same reason, flavonoid extracts presented much higher values for TPC than for the sum of the integrated peaks. Precisely, TPC varied between 1.10 mg FA/g for Kalatur and 2.24 ma FA/g for Armorik, while HPLC sum of the areas ranged from 28.04 μg/g for Ggolden to 87.52 μg/g for Huggo. In this case, the peaks that could be integrated were only 4, compared to the many more detected and tentatively identified (Figure 7). Overall, the levels of flavonoids found in the Austrian varieties studied in the present research were in the range of previously reported values for sorghum (Dykes et al., 2009; Dykes et al., 2011; Su et al., 2017).

The data from the HPLC allowed to tentatively identify unknown peaks based on UV data and retention times reported in literature. In regard to the phenolic fraction, peak 9 was only extracted in free form (Figure 5). It presented a UV λ max at 325 nm and had a comparable spectrum to the one from chlorogenic acid reported in literature (Robbins, 2003). This compound was also found in sorghum in previous studies, with a similar retention behavior (Ghinea et al., 2021). Moreover, a study on millet reported its presence only in a free form (Chandrasekara and Shahidi, 2011). Peaks 10 and 11 were only present in the conjugated phenolic acid fraction and were only detected at 320 nm (Figure 5). Peak 10 eluted just before peak 11 at 19.1 min. The UV spectrum of this compound showed maximum absorbance at 317 nm. By comparing λ max and the characteristics of the spectrum with the data found in previous publications, this compound was tentatively identified as 4-methoxycinnamic acid (Holser, 2008; Xiong et al., 2020). The λ max of the UV spectrum of compound 11 was at 322 nm. By comparison with the data in literature, peak 11 was tentatively identified as sinapic acid (Cai and Arntfield, 2001; Salum et al., 2015; Adelakun and Duodu, 2017). This hypothesis was also supported by the fact that sinapic acid was reported in sorghum in previous studies (Waniska et al., 1989; Dykes and Rooney, 2006) and mainly present in the conjugated fraction (Li et al., 2008; Chandrasekara and Shahidi, 2011). Unknown peaks in the flavonoid chromatograms were also tentatively identified based on UV data and retention times reported in the literature. Specifically, the two major peaks appearing at 480 nm (Figure 7) were identified as 5-MetO-Lut and 7-MetO-Api (Taleon et al., 2014; Su et al., 2017). Peak h was hypothesized to be a phenolic acid, based on the UV spectrum resembling the one from ferulic acid and caffeic acid. Peaks i and j had the same UV spectrum, with maximum absorbance at 271 and 332 nm. Based on this information and their retention time, these peaks could be apigenin glycosides (Xiong et al., 2020). These compounds were only extracted from the Huggo variety in a significant amount. Other varieties did not present any peak or only slightly above the base line. Compound k was detected only in Huggo and Ggolden varieties. The peak height for Ggolden variety was six times smaller than that for Huggo. The UV spectrum of compound l presented maxima at 267 and 327 nm, similarly to apigenin-O-glucoside identified in previous studies (Olennikov et al., 2018).

The antioxidant activity values measured as the capacity to scavenge DPPH and ABTS radicals reported in the literature for white and red sorghum varieties are wide-ranging, between approximately 2 and 200 μmol TE/g (Dykes et al., 2005; Shen et al., 2018). This great range is likely due to differences in the assay protocols among each study. The values obtained for the eight Austrian grown sorghum varieties were on the lower end of the spectrum, between 1.5 and 6.0 μmol TE/g for both DPPH and ABTS. In this study, antioxidant capacity (ABTS and DPPH) had a significant and positive correlation with TPC. Previous studies conducted on several varieties of sorghum also showed a significant correlation between antioxidant assays (Awika et al., 2003; Dykes et al., 2005; Luthria and Liu, 2013).

In conclusion, efficient and effective microscale methods for the extraction of phenolic acids and flavonoids from sorghum were developed. Using these, eight sorghum varieties grown in Austria were screened for antioxidant content and activity. In all sorghum varieties, protocatechuic acid, vanillic acid, caffeic acid, syringic acid, P-coumaric acid, and ferulic acid were detected in free, conjugated, and bound forms, with the latter being the most abundant. The phenolic acid content was between 0.99 and 1.57 mg/g. Also, flavonoids, comprising luteolinidin, apigenidin, naringenin, apigenin, 5-MetO-Lut, and 7-MetO-Api, were detected in amounts between 27.03 and 87.52 μg/g. This study only focused on raw sorghum kernels as a first screening of the antioxidant compounds present in the Austrian grown sorghum varieties. The influence of processing and cooking on sorghum phenolic compounds and antioxidant activity would need to be further investigated as sorghum might find space in the western diet in the future.

## Data Availability Statement

The original contributions presented in the study are included in the article/Supplementary Material, further inquiries can be directed to the corresponding author/s.

## Author Contributions

SS: practical trials, method development, project planning, and manuscript writing. RK: practical trials, method development, and manuscript revision. RW: method development and practical trials. RS: project planning, project leader, manuscript writing and revision, and funding. All authors contributed to the article and approved the submitted version.

## Conflict of Interest

The authors declare that the research was conducted in the absence of any commercial or financial relationships that could be construed as a potential conflict of interest.

## Publisher’s Note

All claims expressed in this article are solely those of the authors and do not necessarily represent those of their affiliated organizations, or those of the publisher, the editors and the reviewers. Any product that may be evaluated in this article, or claim that may be made by its manufacturer, is not guaranteed or endorsed by the publisher.
