# Supplementary material for: Reversed-Phase HPLC Characterization and Quantification and Antioxidant Capacity of the Phenolic Acids and Flavonoids Extracted From Eight Varieties of Sorghum Grown in Austria
Source: Front Plant Sci. 2021 Nov 5;12:769151. doi: 10.3389/fpls.2021.769151 (PMC8604811; doi:10.3389/fpls.2021.769151)
Supplement: Supplementary file 1 [file Data_Sheet_1.docx]

Table S 1. Names and pericarp color of the eight varieties of sorghum used for the study. The moisture % reported here was used for the calculation of results on dry matter. Kernel length was calculated as the average of three kernels per each variety.

| **Variety name** | **Pericarp color** | **Moisture (%)** | **Average kernel length (mm)** | |
| --- | --- | --- | --- | --- |
| **Arabesk** | White | 9.33 | 4.5 ± 0.5 |  |
| **Armorik** | Red | 9.33 | 4.0 ± 0.5 |  |
| **Arsky** | Orange | 9.36 | 4.7 ± 0.2 |  |
| **Ggolden** | White | 9.33 | 3.9 ± 0.4 |  |
| **Huggo** | Red | 10.02 | 4.5 ± 0.4 |  |
| **Icebergg** | White | 9.33 | 4.0 ± 0.7 |  |
| **Kalatur** | White | 9.41 | 4.3 ± 0.4 |  |
| **PR88Y92** | White | 9.25 | 4.2 ± 0.2 |  |


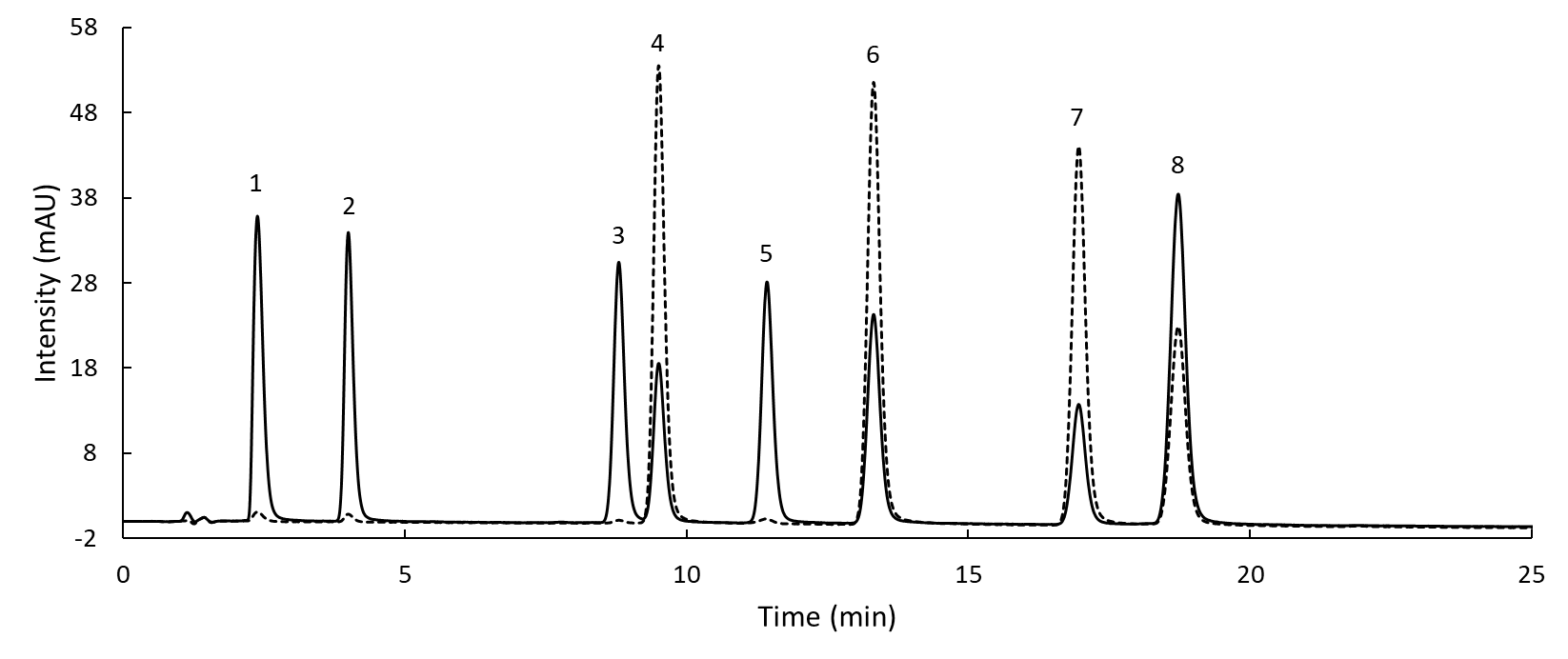


Figure S 2. Chromatogram of the phenolic standards mix (10 µg/ml) recorded at 268 nm (solid line) and at 320 nm (dash line). 1: gallic acid, 2: protocatechuic acid, 3: vanillic acid, 4: caffeic acid, 5: syringic acid, 6: p-coumaric acid, 7: ferulic acid, 8: o-coumaric acid.


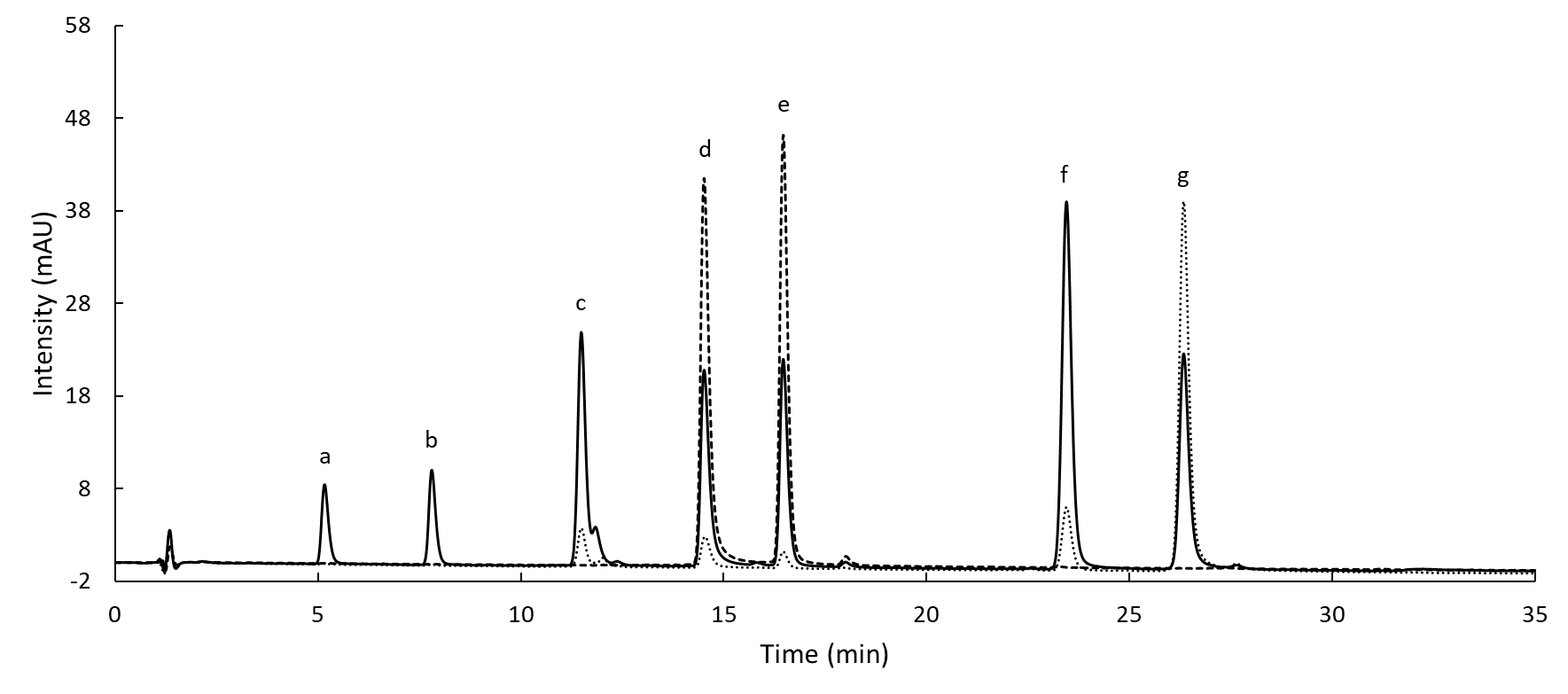


Figure S 3. Chromatogram of the flavonoid standards mix (10 µg/ml) recorded at 280 nm (solid line) and at 340 nm (dot line) and at 480 nm (dash line). a: catechin, b: epicatechin, c: taxifolin, d: luteolinidin-chloride, e: apigenidin-chloride; f: naringenin, g: apigenin.


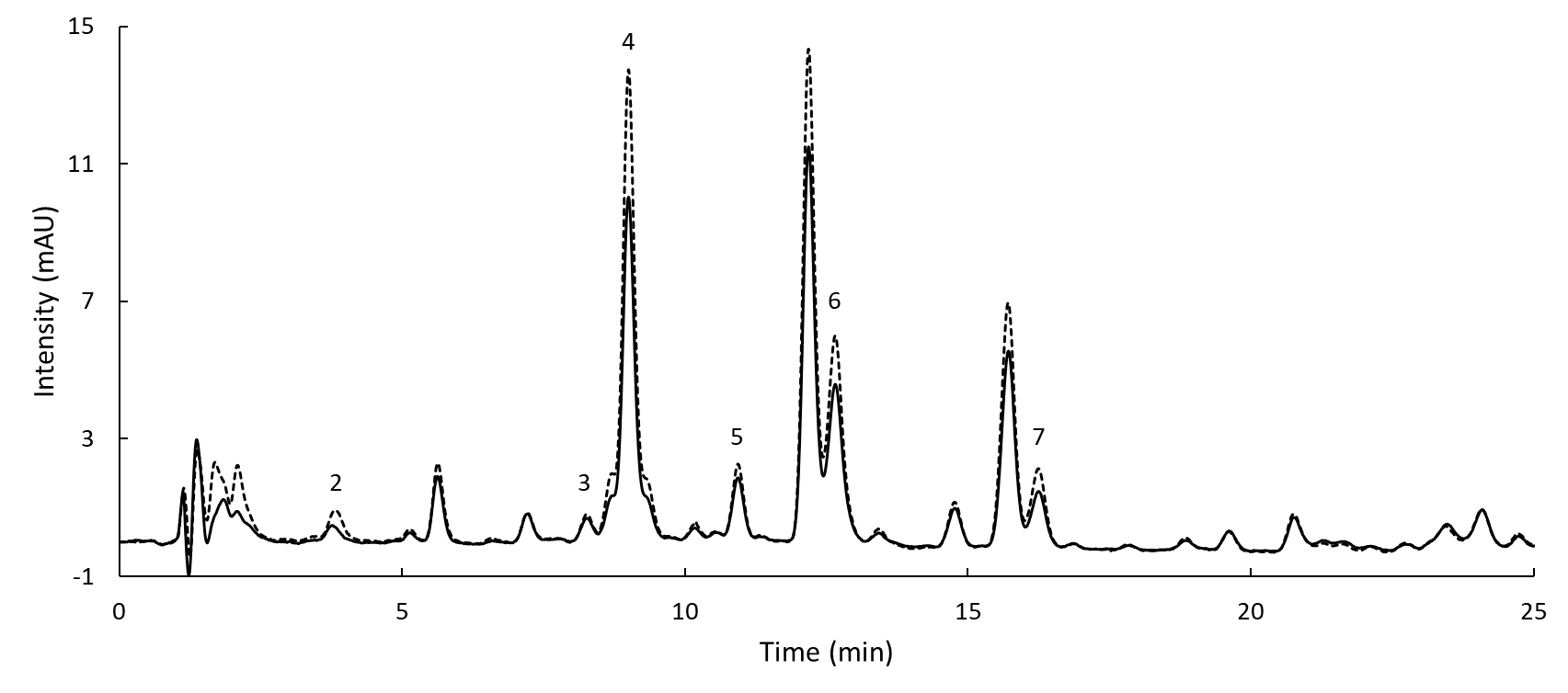


Figure S 4. Chromatogram of the free phenolic fraction of Armorik variety, extracted with ethanol 80 % (Method 3, solid line) and with methanol 80 % (Method 4, dash line), recorded at 268 nm. 2: protocatechuic acid, 3: vanillic acid, 4: caffeic acid, 5: syringic acid, 6: p-coumaric acid, 7: ferulic acid. Injection volume was 10 µl.


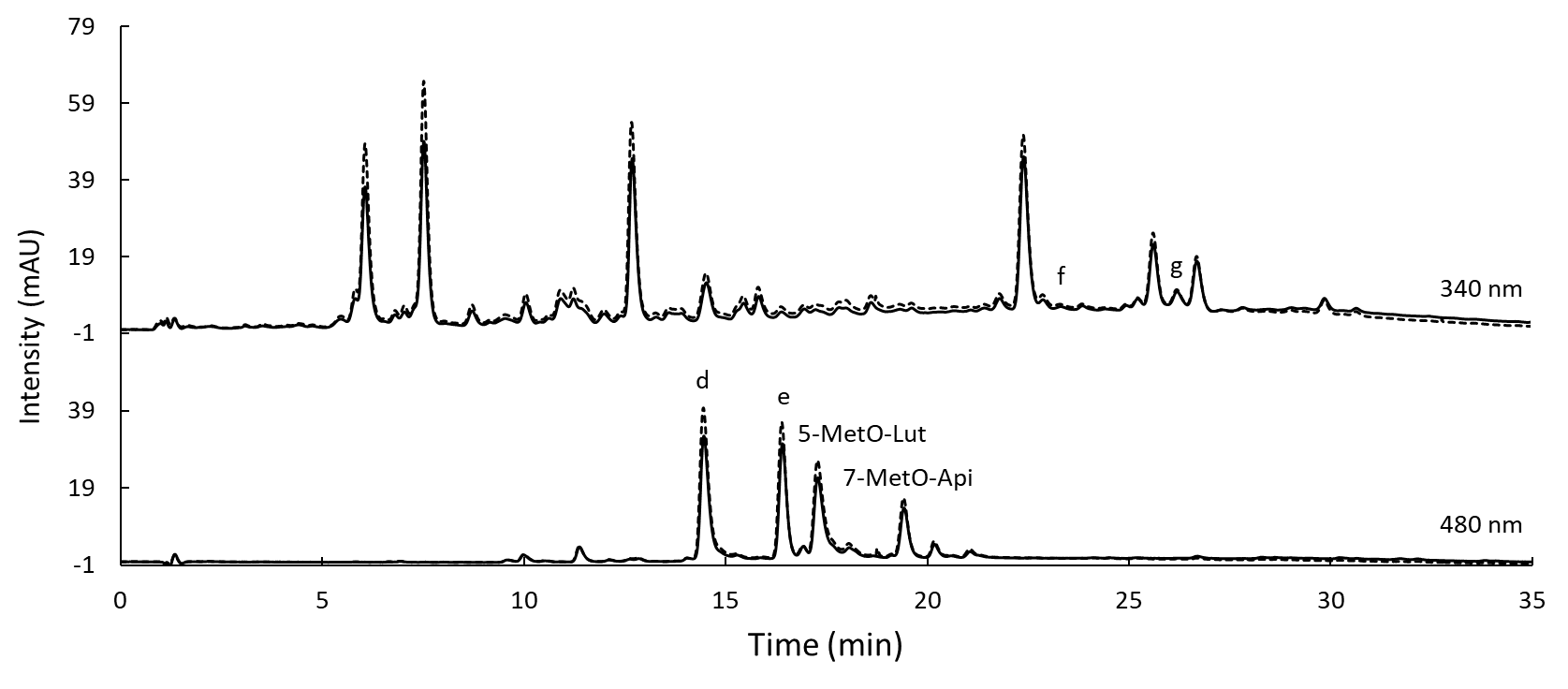


Figure S 5. Chromatogram of the flavonoids extracted from Armorik variety with sonication (Method E, solid line) and with shaking (Method F, dash line) recorded at 340 nm (above) and 480 nm (below). d: luteolinidin-chloride, e: apigenidin-chloride; f: naringenin, g: apigenin. Injection volume: 10 µl.

Table S 2. Sum of the free, conjugated and bound phenolic acids in the eight varieties of sorghum calculated from the peak areas of the HPLC chromatograms. Total phenolic acids (PA) was calculated as sum of the 6 compounds. Results are expressed as mean value ± standard deviation (n=3). The superscript indicates if samples are significantly different (different letter). All results are expressed on dry matter and µg/g.

|  | Protocatechuic acid | Vanillic acid | Caffeic acid | Syringic acid | P-Coumaric acid | Ferulic acid | Total PA |
| --- | --- | --- | --- | --- | --- | --- | --- |
| Arabesk | 9.92 ± 0.60^c,d^ | 11.19 ± 0.96^a^ | 49.68 ± 13.64^a,b,c^ | 3.38 ± 0.21^c,d^ | 86.68 ± 6.18^c,d,e^ | 836.41 ± 57.61^a^ | 997.25 ± 76.05^a,b^ |
| Armorik | 10.87 ± 0.41^d,e^ | 11.25 ± 0.20^a^ | 69.08 ± 2.58^d^ | 4.45 ± 0.03^e^ | 74.71 ± 3.30^b,c^ | 972.68 ± 30.92^a,b^ | 1143.04 ± 36.24^b,c^ |
| Arsky | 8.01 ± 0.57^a,b^ | 13.08 ± 0.58^b,c^ | 40.52 ± 2.49^a^ | 2.08 ± 0.12^a^ | 60.06 ± 6.78^a^ | 798.68 ± 160.03^a^ | 922.65 ± 168.20^a^ |
| Ggolden | 7.80 ± 1.64 ^a^ | 13.59 ± 1.48^b,c^ | 50.35 ± 7.63^a,b,c^ | 3.58 ± 0.10^d^ | 62.93 ± 6.93^a,b^ | 973.78 ± 95.97^a,b^ | 1112.02 ± 100.14^b,c^ |
| Huggo | 11.83 ± 0.89^e^ | 17.30 ± 1.54^d^ | 58.83 ± 3.58^c,d^ | 3.25 ± 0.18^c,d^ | 90.65 ± 11.17^d,e^ | 858.43 ± 155.56^a^ | 1036.49 ± 159.25^a,b^ |
| Icebergg | 11.72 ± 1.17^e^ | 13.34 ± 0.94^b,c^ | 55.68 ± 11.72^b,c,d^ | 3.06 ± 0.24^c^ | 84.98 ± 1.87^c,d^ | 1104.34 ± 74.66^b^ | 1269.28 ± 60.23^c^ |
| Kalatur | 9.14 ± 1.09^a,b,c^ | 14.70 ± 1.23^c^ | 39.97 ± 8.80^a^ | 3.33 ± 0.37^c,d^ | 68.35 ± 13.55^a,b^ | 871.11 ± 126.51^a^ | 1006.60 ± 102.61^a,b^ |
| PR88Y92 | 9.44 ± 0.08^b,c,d^ | 12.82 ± 0.27^a,b^ | 43.77 ± 0.98 1^a,b^ | 2.68 ± 0.09^b^ | 98.49 ± 4.83^e^ | 914.36 ± 82.78^a^ | 1081.56 ± 87.16^a,b^ |
